# Osteoarthritis, Exercise, and Tissue Engineering: A Stimulating Triad for Health Professionals

**DOI:** 10.1155/2019/1935806

**Published:** 2019-05-02

**Authors:** Pedro Morouço, Cristiana Fernandes, Rita Santos-Rocha

**Affiliations:** ^1^Polytechnic Institute of Leiria, School of Education and Social Sciences (ESECS), Leiria, Portugal; ^2^Universidade de Lisboa, Faculdade de Motricidade Humana, Laboratory of Biomechanics and Functional Morphology, Interdisciplinary Centre for the Study of Human Performance (CIPER), Cruz Quebrada, Portugal; ^3^Polytechnic Institute of Leiria, Centre for Rapid and Sustainable Product Development (CDRsp), Marinha Grande, Portugal; ^4^Polytechnic Institute of Santarém, Sport Sciences School of Rio Maior (ESDRM), Rio Maior, Portugal

## Abstract

Osteoarthritis (OA) is a degenerative disease, promoted by abnormal chronic mechanical loading over the joint, for instance, due to excessive body mass. Patients frequently report pain, fatigue, and limitations in specific functional daily activities. Regarding the treatment of OA, two nonpharmacological options are available. However, it is not clear which type and intensity of exercise have better outcomes in treatment and how tissue engineering can be a promising field due to the mechanical load implants will suffer. The aims of this work were to investigate (1) the main characteristics, prevalence, and consequences of OA; (2) the exercise prescription guidelines and whether exercise interventions have a positive effect on OA treatment; and (3) the novel improvements on tissue engineering for OA treatment. Both patients and practitioners should be aware that benefits may come from prescribed and supervised exercise. Recent studies have highlighted that an optimal balance between exercise and nutritional income should be widely recommended. Regarding tissue engineering, significant steps towards the development of implants that mimic the native tissue have been taken. Thus, further studies should focus on the impact that exercise (repetitive loading) might have on cartilage regeneration. Finally, suggestions for future research were proposed.

## 1. Introduction

Osteoarthritis (OA) is a degenerative disease, promoted by abnormal chronic mechanical loading over the joint, for instance, due to excessive body mass [1]. Patients frequently report pain, fatigue, and limitations in specific functional activities. Thus, exercise should be a clear recommendation for OA prevention, but it is not clear which type and intensity [2]. It will increase the load over the joints, and if there is joint malalignment, it will be worse than better [3]. On the other hand, recent improvements in tissue engineering have demonstrated the suitability of novel bilayer scaffolds [4]. However, it is unclear how these scaffolds will behave responding to the normal mechanical loading over time. Moreover, it is demonstrated that mechanical loading is an adequate stimulus for cartilage regeneration [5, 6]. Therefore, it is unclear how these scaffolds will behave responding to abnormal (e.g., resistance training) mechanical loading over time. It may be questioned if a patient treated with one of the mentioned matrices (e.g., MaioRegen®) should be enrolled in exercise to decrease his/her body mass; if yes, with which type of exercise. Several issues demonstrate the stimulating triad that researchers should look up to for promising treatments of osteochondral defects.

**Figure 1:**
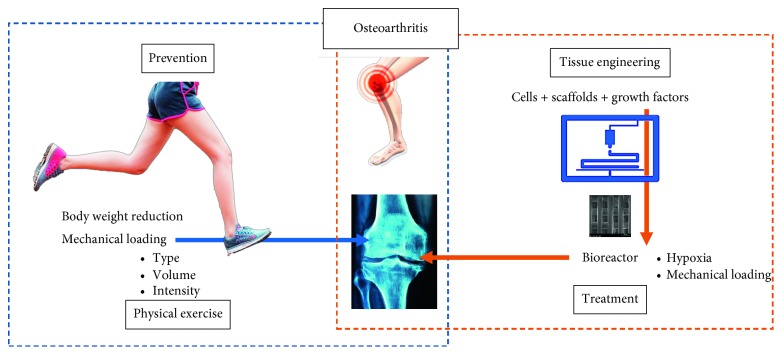
Illustrative diagram of the relationships between osteoarthritis, physical exercise, and tissue engineering.

We do believe that the scientific communities around rheumatic disorders should work together and, more importantly, know what other disciplines are advancing. Physical exercise specialists will soon have clients treated with bioengineered implants, and bioengineers should pay attention to the repetitive load that a scaffold must stand for, even after implanted. Our aim is to foster the potential multidiscipline approach regarding this health issue (Figure 1).

The focus of this manuscript was on the close relationship between OA, exercise, and tissue engineering. The aims were to investigate (1) the main characteristics, prevalence, and consequences of OA; (2) the exercise prescription guidelines, and whether exercise interventions have a positive effect on OA treatment; and (3) the novel improvements on tissue engineering in the treatment of OA.

## 2. Main Characteristics, Prevalence, and Consequences of Osteoarthritis

Cartilage is a tissue with enormous complexity that is found in the human body in three types: hyaline cartilage, fibrocartilage, and elastic cartilage. The articular cartilage is a flexible connective tissue that aligns the surface of the bones in the synovial joints throughout the body, allowing a movement with almost zero friction on its surface. The extracellular matrix is stratified into four distinct (architecturally and biochemically) zones (the surface zone, the midzone, the deep zone, and the calcified zone), which together give rise to its viscoelastic properties [4, 7]. It is avascular, alymphatic, and aneural and, therefore, has a very low endogenous regeneration capacity [8]. Either way, its structure and mechanical properties allow it to handle with repetitive load forces over decades. Thus, damage caused to the joints by trauma or disease usually requires exogenous intervention to stimulate regeneration [9, 10].

According to the World Health Organization, cartilage-related diseases are one of the major societal challenges (Table 1). The prevention of joint cartilage degeneration is an important health issue with a significant number of repair strategies to treat an articular cartilage injury, some already available and others in an on-going research status [11–17]. OA is a heterogeneous group of joint disorders, which may be categorized as primary (idiopathic) or secondary. While primary OA can be defined as a process occurring with an absence of an obvious underlying abnormality, secondary OA is often the result of injury (trauma) or repetitive motion such as found in certain occupations [18]. Either way, the distinct categories do not alter the clinical practice and therapeutic choice. Nevertheless, there are known risk factors, such as overweight or obesity, history of joint injury or surgery, genetic predisposition, and aging [19]. OA is characterized by joint pain and stiffness, usually associated with degeneration of the joint cartilage, commonly in hands, hips, spine, and knees [20]. It is estimated that 18% of women and 9.6% of men over 60 years suffer from symptomatic OA [21]. Moreover, 25% to 50% with OA will have symptoms [20]. In addition, 80% of people with OA will have movement limitations and 25% will not be able to perform daily life activities. This pathology is associated with factors such as aging, obesity, nutritional deficiencies, and physical (in)activity; so that, more than 250 million people are affected with chronic OA.

Aging has been connected to chronic low-grade inflammation, which is sometimes termed inflammation [22]. These changes resulting from age compromise the effectiveness of cartilage repair, which contributes to an increased incidence of OA [19, 23]. As a person ages, there is an increase in fat mass. It happens because there is an increase in the number of proinflammatory adipocytes and macrophages in the adipose tissue that produce cytosines and adipokines [24, 25], as well as fibroblast and vascular endothelial growth factors [26]. It is thus admitted that an increase in the age-related fat volume will contribute to OA. For instance, a study on fat-conditioned medium demonstrated that removing the infrapatellar fat pads from the terminal knee made it become protective [27]. Accordingly, it is critical to come up with more research to underline the mechanisms related to the delay in the loss of function in more than one system [19]. In the 90s, Volpin et al. [28] and Honkonen [29] studied the influence of age on the risk of developing posttraumatic OA and showed that OA increases 3 to 4 times after age 50 [23]. However, more recently, due to the cotemporaneous lifestyle, the incidence of OA in early ages has been increasing [30]. This incidence clearly justifies the need to carry out research that contributes to the weakening of the problem through the interventionist, integrated and multidisciplinary approaches.

From our perspective, regarding the aim of achieving significant inputs to overcome, or at least minimize this societal problem, two interdependent main topics should be addressed: prevention and treatment.

## 3. Exercise for the Prevention of Osteoarthritis

Although mechanical stimulation plays a vital role in maintaining cartilage homeostasis, excessive loading is a known contributor to the development of degenerative joint diseases, such as OA. It is characterized by damaging the articular cartilage with the development of osteophytes and inflammation of the synovial membrane [36]. Accordingly, excessive mechanical compression may induce degradation of the matrix. That is why OA is strongly associated with mechanical risk factors, such as obesity, joint overload, or injury, making it relevant data for prevention [37]. Therefore, the development of adequate exercise programs for an elderly population (a high-risk population) and its massive dissemination is mandatory [38]. Different types of exercises (e.g., resistance training and water-based workout) may induce significant effects in the prevention of this pathology [39, 40].

It should be highlighted that studies have shown that most exercise does not aggravate the symptoms nor increase the progression of arthritis [20]. Indeed, health professionals should embrace the idea that exercise is not only safe but also generally reported to reduce pain, fatigue, inflammation, and disease activity. For instance, both high- (i.e., running) and low- (i.e., walking) intensity aerobic exercises appear to be effective in improving functional status, gait, pain, and aerobic capacity [41]. Moreover, resistance training and flexibility exercises are also important, as well as incorporating functional exercises to improve neuromuscular control, balance, and ability to perform activities of daily living [20]. Although it is well accepted that exercise maintains and improves strength and aerobic capacity [41], the results available in the literature are inconclusive. Minimizing or preventing functional decline attenuates pain and joint stiffness and aids in weight control [20, 38, 42]. Still, the development of new investigations (through randomized controlled trials), clarifying the most adequate prescription according to the stage of OA, is crucial [41, 43].

The tolerance volume may vary from one day to the next, which will imply flexibility in activities and exercise options. Either way, the human body is as a well-established sensorial network to guide us. When the joints are sore and inflamed, intensity should be reduced to keep the load within its tolerance. As rule of thumb, a meta-regression analysis stated that focus should be on improving aerobic capacity, quadriceps muscle strength, or lower extremity performance, and the program should be supervised and carried out 3 times a week [44]. As well as, to maintain healthy joints, a proper balance of the amount and type of exercise is necessary. The development of structured exercises should provide enough options to allow the patient to flex the activity options and to maintain the load within the joint with different training parameters. This can differentiate between the amount of weight used, the volume of repetition, and among others [9]. Most important, the patient should like and be motivated for the proposed activities. Otherwise, his/her engagement will be insufficient to obtain the intended results. Exercise prescription should take into consideration the individual's disease activity, pain, functional limitations, and personal preferences to optimize adoption and adherence to exercise [20]. Special considerations should include avoiding strenuous exercises during acute flare-ups; advising that small amount of discomfort in the muscles or joints during or immediately after exercise is common following performance of unfamiliar exercise; substituting the program with alternative exercises when specific exercises exacerbate joint pain; and wearing appropriate shoes that provide good shock absorption and stability are equally important [20, 45].

Likewise, both patients and practitioners should be aware that benefits may come from prescribed and supervised exercise, but also from a more active daily lifestyle [46–48].

## 4. Tissue Engineering for the Treatment of Osteoarthritis

Tissue engineering (TE) emerged in the 1980s with a colossal potential due to the complexity of human tissues. The main goal of TE is to develop biological substitutes that restore, maintain, or enhance the function of tissues and organs based on materials engineering and life sciences [49]. Its main challenge is to provide an adequate function according to the tailored structure. In fact, choosing the right approach to tissue regeneration is a huge concern for all researchers in this field.

The replacement of tissues (such as bone or cartilage) or joints with allograft materials includes the risk of infections by viruses (such as HIV and hepatitis C), graft vs. host disease [50] or even, chondrocytes can die during the process [51]. Also, the use of grafts can only be applied to damaged areas of less than 2 cm^2^ [15]. Accordingly, researchers have been interested in developing alternative approaches for restoring joint functionality, which can be translated to clinical practice. Although there has been an enormous amount of work with the goal of regenerating cartilage, a personalized construction has not yet been achieved and disseminated. Cartilage has a major role in providing joints with a surface that combines low friction with high lubrication [52]; thus, a deeper knowledge on cartilage characterization, bridging the gap between anatomy and physiology, may lead the way for better implants aiming cartilage repair and regeneration [53].

The regeneration of articular cartilage resulting from degenerative joint disease, such as OA, is an emerging area under investigation using TE approaches. Recent investigations highlighted promising regenerative strategies [37, 54]. For instance, the implantation of an autologous chondrocytes matrix and the immunization of autologous chondrocytes promise high potential for the regeneration of hyaline cartilage [37]. Some strategies are already available on the market and others under investigation: palliative; microfracture grafts; cell-based; whole tissue transplantation; scaffold-based or cell plus scaffold-based; and hydrogels-based or cell plus hydrogel-based. Accordingly, we might question that why is OA a burden health problem? Probably, because there is a lack of customization on the used approaches; because experiments should look for higher reliability; and because randomized controlled trials are needed to bridge the gap between the labs and the clinicians.

For instance, not long ago, it was reported that most commonly used three-dimensional (3D) scaffold architectures in cartilage TE were porous 3D sponges [55]. This nonconventional procedure does not allow control over the inner architecture, thus, not guaranteeing the desired interconnectivity between pores. Embracing technology advancements, TE overcomes some of the mentioned drawbacks, in particular providing a customized design [56]. Additive manufacturing (AM), also known as 3D printing, technologies allow the production of complex 3D structures with a high level of control, predefined geometry, size, and interconnected pores, in a reproducible way [57]. This controlled organization enhances the vascularization and, thus, transport of oxygen and nutrients throughout the whole structure, providing an adequate biomechanical environment for tissue regeneration [58]. The recent advancements in AM have permitted the design and fabrication of patient-specific scaffolds that possess structural and functional features comparable to the native tissue. It allows for design and fabrication using tissue images captured with commonly used medical imaging techniques such as computer tomography (CT) and magnetic resonance imaging (MRI) that are readily available in hospitals, something that conventional fabrication techniques lack.

Recently, hydrogel scaffolds have been developed [59]. These hydrogels are designed to provide cells with a fully hydrated 3D environment, comparable to the native tissue extracellular matrix. However, hydrogels have inadequate mechanical properties that are unfavorable for embedded cells or become too weak for application to the musculoskeletal system [60]. Accordingly, one promising strategy is to fabricate reinforced implants combining printed microfibers with hydrogels [61]. Or more recently, having an in situ approach with a biopen to repair a full‐thickness chondral defect [62] or a 4D bioprinting technology that fabricates dynamic structures improves the cartilage and bone regeneration [63, 64]. Undoubtedly, significant efforts are being developed worldwide in the fields of tissue engineering and regenerative medicine, but full osteochondral restoration remains a paramount challenge; it should not be forgotten that OA is much more than a failure in the cartilage. Total contemplation over osteochondral defects should be considered: anatomy, structure and composition resembling native tissue; biomechanics, ability to yield similar mechanical behavior; and physiology, fully restore joint functionality [65]. The different compositions and mechanical properties of bone and cartilage indicate the complexity of this tissue interface, making it challenging for the design and fabrication of tissue engineered scaffolds (for more details, we have recently published a review on biofabrication for osteochondral tissue [4]).

## 5. Conclusion

OA remains in constant discussion and evolution, as it continues to be a challenging, frustrating, and costly problem. It is not only vital to understand the science behind the structure and function of articular cartilage components but also how they interact with risk factors. In this way, health professionals should act preventively, with adequate choices of lifestyles for the long-term health of their patient's joints, as well as contributing to the optimization and customization of treatment by TE.

## Figures and Tables

**Table 1:** Top facts related to OA.

(i) Disability due to musculoskeletal disorders increased by 45% from 1990 to 2010 [31]
(ii) OA is the fastest increasing health condition, affecting over 250 million people worldwide [32]
(iii) It is not exclusive for the elderly: more than 50% of adults with knee OA are under 65 [30]
(iv) Two out of three people with obesity are at risk for developing knee OA in their lifetime [33]
(v) Knee OA is the 11th leading cause of disability and shows a growing trend [34]
(vi) People with OA have a 16% increased risk of developing cardiovascular disease compared to those without [35]

## References

[1] Griffin T. M., Guilak F. (2005). The role of mechanical loading in the onset and progression of osteoarthritis. *Exercise and Sport Sciences Reviews*.

[2] Leong D. J., Sun H. B. (2014). Osteoarthritis-why exercise?. *Journal of Exercise, Sports & Orthopedics*.

[3] Sharma L., Dunlop D. D., Cahue S., Song J., Hayes K. W. (2003). Quadriceps strength and osteoarthritis progression in malaligned and lax knees. *Annals of Internal Medicine*.

[4] Abdulghani S., Morouço P. G. (2019). Biofabrication for osteochondral tissue regeneration: bioink printability requirements. *Journal of Materials Science: Materials in Medicine*.

[5] Elder B. D., Athanasiou K. A. (2009). Hydrostatic pressure in articular cartilage tissue engineering: from chondrocytes to tissue regeneration. *Tissue Engineering Part B: Reviews*.

[6] Madeira C., Santhagunam A., Salgueiro J. B., Cabral J. M. S. (2015). Advanced cell therapies for articular cartilage regeneration. *Trends in Biotechnology*.

[7] Sophia Fox A. J., Bedi A., Rodeo S. A. (2009). The basic science of articular cartilage: structure, composition, and function. *Sports Health: A Multidisciplinary Approach*.

[8] Natoli R. M., Revell C. M., Athanasiou K. A. (2009). Chondroitinase ABC treatment results in greater tensile properties of self-assembled tissue-engineered articular cartilage. *Tissue Engineering Part A*.

[9] Brody L. T. (2015). Knee osteoarthritis: clinical connections to articular cartilage structure and function. *Physical Therapy in Sport*.

[10] Lam J., Lee E. J., Clark E. C., Mikos A. G. (2017). Honing cell and tissue culture conditions for bone and cartilage tissue engineering. *Cold Spring Harbor Perspectives in Medicine*.

[11] Toh W. S., Spector M., Lee E. H., Cao T. (2011). Biomaterial-mediated delivery of microenvironmental cues for repair and regeneration of articular cartilage. *Molecular Pharmaceutics*.

[12] Williams R. J., Brophy R. H. (2007). Decision making in cartilage repair procedures. *Cartilage Repair Strategies*.

[13] Sargeant T. D., Desai A. P., Banerjee S., Agawu A., Stopek J. B. (2012). An in situ forming collagen-PEG hydrogel for tissue regeneration. *Acta Biomaterialia*.

[14] Bhosale A. M., Richardson J. B. (2008). Articular cartilage: structure, injuries and review of management. *British Medical Bulletin*.

[15] Cancedda R., Dozin B., Giannoni P., Quarto R. (2003). Tissue engineering and cell therapy of cartilage and bone. *Matrix Biology*.

[16] Williams R. J., Niederauer G. G. (2007). Articular cartilage resurfacing using synthetic resorbable scaffolds. *Cartilage Repair Strategies*.

[17] Söntjens S. H. M., Nettles D. L., Carnahan M. A., Setton L. A., Grinstaff M. W. (2006). Biodendrimer-based hydrogel scaffolds for cartilage tissue repair. *Biomacromolecules*.

[18] Samson D. J., Grant M. D., Ratko T. A., Bonnell C. J., Ziegler K. M., Aronson N. (2007). Treatment of primary and secondary osteoarthritis of the knee. *Evidence Report/Technology Assessment*.

[19] Loeser R. F., Collins J. A., Diekman B. O. (2016). Ageing and the pathogenesis of osteoarthritis. *Nature Reviews Rheumatology*.

[20] Medicine AC of S (2017). *ACSM’s Guidelines for Exercise Testing and Prescription*.

[21] Murray C. J. L., Lopez A. D., Organization W. H. (1996). *The Global Burden of Disease: a Comprehensive Assessment of Mortality and Disability from Diseases, Injuries, and Risk Factors in 1990 and Projected to 2020: Summary*.

[22] Franceschi C., Bonafè M., Valensin S. (2000). Inflamm-aging. An evolutionary perspective on immunosenescence. *Annals of the New York Academy of Sciences*.

[23] Brittberg M., Spector M., Farr J. (2016). Cellular senescence in aging and osteoarthritis. *Acta Orthopaedica*.

[24] Sellam J., Berenbaum F. (2013). Is osteoarthritis a metabolic disease?. *Joint Bone Spine*.

[25] Guilak F. (2011). Biomechanical factors in osteoarthritis. *Best Practice & Research Clinical Rheumatology*.

[26] Ushiyama T., Chano T., Inoue K., Matsusue Y. (2003). Cytokine production in the infrapatellar fat pad: another source of cytokines in knee synovial fluids. *Annals of the Rheumatic Diseases*.

[27] Clockaerts S., Bastiaansen-Jenniskens Y. M., Bridts C. (2011). Infrapatellar fat pad of patients with end-stage osteoarthritis inhibits catabolic mediators in cartilage. *Annals of the Rheumatic Diseases*.

[28] Volpin G., Dowd G., Stein H., Bentley G. (1990). Degenerative arthritis after intra-articular fractures of the knee. Long-term results. *The Journal of Bone and Joint Surgery. British Volume*.

[29] Honkonen S. E. (1995). Degenerative arthritis after tibial plateau fractures. *Journal of Orthopaedic Trauma*.

[30] Deshpande B. R., Katz J. N., Solomon D. H. (2016). Number of persons with symptomatic knee osteoarthritis in the US: impact of race and ethnicity, age, sex, and obesity. *Arthritis Care & Research*.

[31] Vos T., Flaxman A. D., Naghavi M. (2012). Years lived with disability (YLDs) for 1160 sequelae of 289 diseases and injuries 1990–2010: a systematic analysis for the global burden of disease study 2010. *Lancet*.

[32] Hunter D. J., Schofield D., Callander E. (2014). The individual and socioeconomic impact of osteoarthritis. *Nature Reviews Rheumatology*.

[33] Murphy L., Schwartz T. A., Helmick C. G. (2008). Lifetime risk of symptomatic knee osteoarthritis. *Arthritis & Rheumatism*.

[34] Lohmander L. S. (2013). Knee replacement for osteoarthritis: facts, hopes, and fears. *Medicographia*.

[35] Williams A., Kamper S. J., Wiggers J. H. (2018). Musculoskeletal conditions may increase the risk of chronic disease: a systematic review and meta-analysis of cohort studies. *BMC Medicine*.

[36] Loeser R. F. (2010). Age-related changes in the musculoskeletal system and the development of osteoarthritis. *Clinics in Geriatric Medicine*.

[37] Fahy N., Alini M., Stoddart M. J. (2017). Mechanical stimulation of mesenchymal stem cells: implications for cartilage tissue engineering. *Journal of Orthopaedic Research*.

[38] Fransen M., McConnell S., Harmer A. R., Van der Esch M., Simic M., Bennell K. L. (2015). Exercise for osteoarthritis of the knee: a Cochrane systematic review. *British Journal of Sports Medicine*.

[39] Yázigi F., Espanha M., Vieira F., Messier S. P., Monteiro C., Veloso A. P. (2013). The PICO project: aquatic exercise for knee osteoarthritis in overweight and obese individuals. *BMC Musculoskeletal Disorders*.

[40] Bartels E. M., Lund H., Hagen K. B., Dagfinrud H., Christensen R., Danneskiold-Samsøe B. (2007). Aquatic exercise for the treatment of knee and hip osteoarthritis. *Cochrane Database of Systematic Reviews*.

[41] Brosseau L., MacLeay L., Robinson V., Wells G., Tugwell P. (2003). Intensity of exercise for the treatment of osteoarthritis (Cochrane Review). *Cochrane Database of Systematic Reviews*.

[42] Fransen M., McConnell S., Hernandez-Molina G., Reichenbach S. (2009). Exercise for osteoarthritis of the hip. *Cochrane Database of Systematic Reviews*.

[43] Waller B., Munukka M., Multanen J. (2013). Effects of a progressive aquatic resistance exercise program on the biochemical composition and morphology of cartilage in women with mild knee osteoarthritis: protocol for a randomised controlled trial. *BMC Musculoskelet Disorders*.

[44] Juhl C., Christensen R., Roos E. M., Zhang W., Lund H. (2014). Impact of exercise type and dose on pain and disability in knee osteoarthritis: a systematic review and meta-regression analysis of randomized controlled trials. *Arthritis & Rheumatology*.

[45] Beckwée D., Vaes P., Cnudde M., Swinnen E., Bautmans I. (2013). Osteoarthritis of the knee: why does exercise work? A qualitative study of the literature. *Ageing Research Reviews*.

[46] Loeser R. F. (2017). The role of aging in the development of osteoarthritis. *Transactions of the American Clinical and Climatological Association*.

[47] Hunter D. J., Beavers D. P., Eckstein F. (2015). The intensive diet and exercise for arthritis (IDEA) trial: 18-month radiographic and MRI outcomes. *Osteoarthritis and Cartilage*.

[48] Yu S. P.-C., Hunter D. J. (2015). Emerging drugs for the treatment of knee osteoarthritis. *Expert Opinion on Emerging Drugs*.

[49] Woodfield T., Lim K., Morouço P., Levato R., Malda J., Melchels F. (2017). 5.14 biofabrication in tissue engineering. *Comprehensive Biomaterials II*.

[50] Pörtner R., Nagel-Heyer S., Goepfert C., Adamietz P., Meenen N. M. (2005). Bioreactor design for tissue engineering. *Journal of Bioscience and Bioengineering*.

[51] Chiang H., Jiang C.-C. (2009). Repair of articular cartilage defects: review and perspectives. *Journal of the Formosan Medical Association*.

[52] Jeffrey D. R., Watt I. (2003). Imaging hyaline cartilage. *The British Journal of Radiology*.

[53] Mouser V. H. M., Levato R., Bonassar L. J. (2017). Three-dimensional bioprinting and its potential in the field of articular cartilage regeneration. *Cartilage*.

[54] Levato R., Webb W. R., Otto I. A. (2017). The bio in the ink: cartilage regeneration with bioprintable hydrogels and articular cartilage-derived progenitor cells. *Acta Biomaterialia*.

[55] Izadifar Z., Chen X., Kulyk W. (2012). Strategic design and fabrication of engineered scaffolds for articular cartilage repair. *Journal of Functional Biomaterials*.

[56] Melchels F. P. W., Domingos M. A. N., Klein T. J., Malda J., Bartolo P. J., Hutmacher D. W. (2012). Additive manufacturing of tissues and organs. *Progress in Polymer Science*.

[57] Morouço P. G. (2018). The usefulness of direct digital manufacturing for biomedical applications. *Intensification of Biobased Processes*.

[58] Zadpoor A. A., Malda J. (2017). Additive manufacturing of biomaterials, tissues, and organs. *Annals of Biomedical Engineering*.

[59] Cao Y., Xiong D., Wang K., Niu Y. (2017). Semi-degradable porous poly (vinyl alcohol) hydrogel scaffold for cartilage repair: evaluation of the initial and cell-cultured tribological properties. *Journal of the Mechanical Behavior of Biomedical Materials*.

[60] Malda J., Visser J., Melchels F. P. (2013). 25th anniversary article: engineering hydrogels for biofabrication. *Advanced Materials*.

[61] Visser J., Melchels F. P. W., Jeon J. E. (2015). Reinforcement of hydrogels using three-dimensionally printed microfibres. *Nature Communications*.

[62] Di Bella C., Duchi S., O’Connell C. D. (2018). In situ handheld three-dimensional bioprinting for cartilage regeneration. *Journal of Tissue Engineering and Regenerative Medicine*.

[63] Li Y.-C., Zhang Y. S., Akpek A., Shin S. R., Khademhosseini A. (2016). 4D bioprinting: the next-generation technology for biofabrication enabled by stimuli-responsive materials. *Biofabrication*.

[64] Gao B., Yang Q., Zhao X., Jin G., Ma Y., Xu F. (2016). 4D bioprinting for biomedical applications. *Trends in Biotechnology*.

[65] Kock L., van Donkelaar C. C., Ito K. (2012). Tissue engineering of functional articular cartilage: the current status. *Cell and Tissue Research*.

